# The Emerging Profile of Cross-Resistance among the Nonnucleoside HIV-1 Reverse Transcriptase Inhibitors

**DOI:** 10.3390/v6082960

**Published:** 2014-07-31

**Authors:** Nicolas Sluis-Cremer

**Affiliations:** Department of Medicine, Division of Infectious Diseases, University of Pittsburgh School of Medicine, S817 Scaife Hall, 3550 Terrace Street, Pittsburgh, PA 15261, USA; E-Mail: nps2@pitt.edu; Tel.: +1-412-648-8457; Fax: +1-412-648-8521

**Keywords:** HIV, reverse transcriptase, nonnucleoside inhibitors, nevirapine, efavirenz, rilpivirine, etravirine, dapivirine, MIV-150

## Abstract

Nonnucleoside reverse transcriptase inhibitors (NNRTIs) are widely used to treat HIV-1-infected individuals; indeed most first-line antiretroviral therapies typically include one NNRTI in combination with two nucleoside analogs. In 2008, the next-generation NNRTI etravirine was approved for the treatment of HIV-infected antiretroviral therapy-experienced individuals, including those with prior NNRTI exposure. NNRTIs are also increasingly being included in strategies to prevent HIV-1 infection. For example: (1) nevirapine is used to prevent mother-to-child transmission; (2) the ASPIRE (MTN 020) study will test whether a vaginal ring containing dapivirine can prevent HIV-1 infection in women; (3) a microbicide gel formulation containing the urea-PETT derivative MIV-150 is in a phase I study to evaluate safety, pharmacokinetics, pharmacodynamics and acceptability; and (4) a long acting rilpivirine formulation is under-development for pre-exposure prophylaxis. Given their widespread use, particularly in resource-limited settings, as well as their low genetic barriers to resistance, there are concerns about overlapping resistance between the different NNRTIs. Consequently, a better understanding of the resistance and cross-resistance profiles among the NNRTI class is important for predicting response to treatment, and surveillance of transmitted drug-resistance.

## 1. Reverse Transcription

Reverse transcription of the single-stranded (+) RNA genome into double-stranded DNA is an essential step in the HIV-1 replication life-cycle [1]. This process is complex and requires the concerted functioning of both the DNA polymerase and ribonuclease H (RNase H) active sites of HIV-1 reverse transcriptase (RT). RT initiates (−) strand DNA synthesis at the 3'end of a cellular lysyl-tRNA^Lys3^ molecule that is hybridized to the primer binding site (PBS) of the viral RNA genome. This nascent DNA strand is elongated by the RNA-dependent DNA polymerase activity (RDDP) of RT until the 5' end of the HIV-1 RNA is reached. RT then uses its RNase H activity to hydrolyze the RNA strand of the RNA/DNA duplex which allows the DNA to hybridize with a repeat sequence at the 3' end of the HIV-1 RNA. Following this strand transfer, the viral DNA strand is elongated by the RDDP activity of RT until the entire RNA template has been copied. The RNase H activity of RT also hydrolyzes the HIV-1 RNA during synthesis of (−) strand DNA, except for a purine rich sequence, termed the polypurine tract (PPT), which serves as a primer for the initiation of (+) strand DNA synthesis. The DNA-dependent DNA polymerase (DDDP) activity of RT elongates the PPT primer. Removal of the PPT and tRNA primers by RT RNase H activity then allows a second strand transfer to take place by interaction of complementary PBS sequences. The HIV-1 RT DDDP activity including strand-displacement activity completes the synthesis of the double stranded proviral DNA precursor.

## 2. Reverse Transcriptase Inhibitors

**Figure 1:**
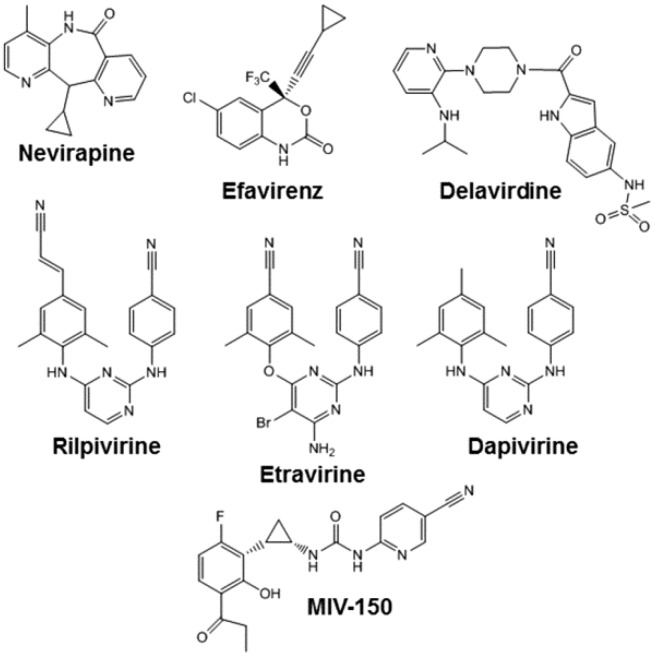
Chemical structures of nonnucleoside reverse transcriptase inhibitors (NNRTIs) used in HIV-1 prevention and treatment strategies.

Due to its essential role in HIV-1 replication, RT is a major target for antiviral drug development and two classes of inhibitors, (1) the nucleoside and nucleotide RT inhibitors (NRTIs) and (2) the nonnucleoside RT inhibitors (NNRTIs), have been approved by the FDA for the treatment of HIV-1 infection. The NRTIs are analogs of naturally occurring dNTPs that lack a 3'-hydroxyl group on the ribose sugar/pseudosugar [2,3]. To exhibit antiviral activity, NRTIs must be metabolically converted by host-cell kinases to their corresponding triphosphate forms, which then inhibit viral DNA synthesis by acting as chain-terminators of DNA synthesis. Eight NRTIs have been approved for clinical use, namely zidovudine, didanosine, zalcitabine, lamivudine, stavudine, abacavir, tenofovir disoproxil fumarate, and emtricitabine. Zalcitabine is less now rarely used to treat HIV-1 infection because it has inconvenient dosing schedules, and is associated with serious adverse events. Similarly, the World Health Organization advocated that stavudine should be phased out of use because of its long-term, irreversible side-effects. In contrast, the NNRTIs are a group of amphiphilic compounds that bind to a hydrophobic pocket in HIV-1 RT that is proximal to but distinct from the polymerase active site (described in more detail below) [2,3]. FDA-approved NNRTIs include nevirapine (NVP), delavirdine, efavirenz (EFV), etravirine (ETV) and rilpivirine (RIL) (Figure 1). The efficacy of delavirdine is lower than that of the other NNRTIs, especially EFV, and it also has an inconvenient dosing schedule. These factors have led the U.S. Department of Health and Human Services (DHHS) Antiretroviral Guidelines Panel to recommend that it not be used as part of initial therapy.

## 3. Mechanism of Action of NNRTIs

NNRTIs interact with HIV-1, but not HIV-2, RT by binding to a single site on the p66 subunit of the HIV-1 RT p66/p51 heterodimer termed the NNRTI binding pocket (NNRTI-BP) that is situated approximately 10 Å from the RT DNA polymerase active site [4]. Crystallographic analyses of HIV-1 RT in complex with NNRTI have suggested at least three possible mechanisms that explain NNRTI inhibition: (1) Esnouf *et al.* reported that NNRTI binding distorts the precise geometry of the DNA polymerase catalytic site, especially the highly conserved tyrosine-methionine-aspartic acid-aspartic acid (YMDD) motif and proposed that this class of drugs inhibits DNA polymerization by locking the polymerase active site in an inactive conformation [5]; (2) Hsiou *et al.* observed that NNRTI binding deformed the structural elements that comprise the “primer grip”, a region in RT that is involved in the precise positioning of the primer DNA strand in the polymerase active site [6]. This change in “primer grip” conformation may alter the position and conformation of the template/primer (T/P) substrate thereby preventing the establishment of a catalytically competent ternary complex; (3) Kohlstaedt *et al.* proposed that the NNRTI-BP may normally function as a hinge between the palm and thumb domains [4]. Since the mobility of the thumb may be important to facilitate T/P translocation, the binding of NNRTIs may restrict the mobility of the thumb domain thereby slowing down or preventing T/P translocation and/or elongation of nascent viral DNA. The three mechanisms suggested above are not mutually exclusive, and NNRTIs may exert multiple inhibitory effects on RT catalyzed DNA synthesis.

## 4. Clinical Use of NNRTIs

**Figure 2:**
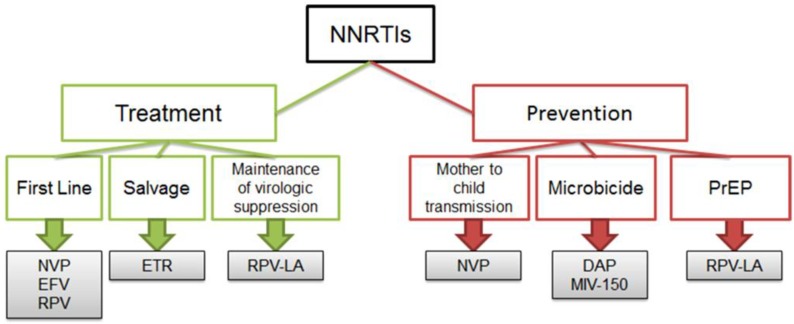
Expanding use of NNRTIs in HIV-1 prevention and treatment strategies.

NNRTIs are widely used to treat HIV-1-infected individuals (Figure 2). Indeed, most first-line antiretroviral therapies (ART) include one NNRTI (typically NVP, EFV or RPV) in combination with two NRTIs. In 2008, ETR was approved for the treatment of HIV-infected ART-experienced individuals, including those with prior NNRTI exposure. NNRTIs are also increasingly being included in strategies to prevent HIV-1 infection (Figure 2). For example: (1) NVP is used to prevent mother-to-child transmission; (2) the ASPIRE (MTN 020) study will test whether a vaginal ring containing the diarylpyrimidine analog dapivirine (DAP; Figure 1) can prevent HIV-1 infection in women; (3) a microbicide gel formulation containing the urea-PETT derivative MIV-150 (Figure 1) is in a phase I study to evaluate safety, pharmacokinetics, pharmacodynamics and acceptability; and (4) a long acting RPV formulation is under-development for pre-exposure prophylaxis (PrEP). As described above, all NNRTIs bind to the same hydrophobic pocket in HIV-1 RT, and all NNRTI-associated resistance mutations are located within, or adjacent to, this pocket. Consequently, there are major concerns about overlapping resistance profiles among the different NNRTIs used for the prevention and treatment of HIV-1 infection. Below, we discuss each of the NNRTIs described above and their resistance profiles.

## 5. Nevirapine

NVP is a dipyridodiazepinone inhibitor [7], and was the first NNRTI approved by the U.S. FDA. At the time it was developed the concept of combination ART had not yet established, and consequently NVP was initially assessed in humans as monotherapy or in combination with zidovudine [8,9]. Needless to say, HIV-1 virologic suppression was transient and plasma viremia returned to pre-treatment levels in a matter of weeks. This rapid loss of activity was associated with the emergence of NVP-resistant virus. The most common mutations associated with NVP montherapy included K103N, V106A, V108I, Y181C, Y188C/H/L and G190A/S/E [8]. When combined with zidovudine, resistance mutations occurred at codons 103, 106 (V106A), 188 and 190, but not at 181 [9]. Subsequent virology and biochemical studies revealed that the Y181C mutation antagonized the thymidine analog mutations associated with zidovudine resistance [10,11]. Currently, the United States Department of Health and Human Services (DHHS) and the International AIDS society (IAS-USA) guidelines recommend NVP as an alternative to EFV for an initial NNRTI-containing regimen in patients who cannot tolerate EFV, are pregnant, or may become pregnant and have fewer than 250 CD4 cells/µL. NVP, in combination with two NRTIs, is also routinely used for fist line therapy in resource limited countries. Interestingly, there is evidence that naturally occurring genetic differences in different HIV-1 subtypes can impact NVP susceptibility and resistance. For example, the V106M RT substitution has been reported to occur more frequently in subtype C viruses than in subtype B [12]. Additionally, mutations in the connection domain of HIV-1 RT, specifically N348I, have also been associated with NVP exposure [13,14]. Typically, N348I emerges at the same time, or after, NNRTI resistance mutations [13,14], but in the context of polymerase domain mutations reduces susceptibility to NVP by 8.9–13-fold [14]. Because it is not teratogenic, NVP is also used for the prevention of mother-to-child transmission in developing countries. However, administration of a single dose of NVP results in the selection of resistance in the treated mothers, which can negatively influence the efficacy of subsequent anti-HIV-1 treatment with NNRTIs [15]. Accordingly, the World Health Organization recommended that in addition to sdNVP, zidovudine monotherapy should be administered to the mother during late gestation, and that short course combination ART “tails”—such as zidovudine and lamivudine, zidovudine and didanosine or tenofovir and emtrictiabine—be administered to the mother and infant to further suppress viral replication and increase the genetic barrier to resistance [15].

## 6. Efavirenz

EFV, a benzoxazinone inhibitor [16], is the most frequently used NNRTI in treatment naïve HIV-infecetd individuals. Its efficacy has been established in numerous clinical trials. For example, studies have compared EFV against protease inhibitors, integrase inhibitors, CCR5 inhibitors, other NNRTIs and triple NRTI regimens. In addition, EFV has been used as the common “third agent” in evaluations of many NRTI combinations. The early randomized, open-label DMP 266-006 study showed that EFV was superior to unboosted indinavir when both were administered over 48 weeks with an NRTI backbone of zidovudine plus lamivudine [17]. In other studies, EFV was as effective as unboosted atazanavir and more effective than unboosted nelfinavir when all were combined with two NRTIs [18,19]. In the 2NN study, NVP and EFV were shown to have similar efficacy [20]. The ACTG A5095 study revealed that virological failure occurred in almost twice as many of the participants treated with the triple NRTI regimen containing zidovudine, lamivudine and abacavir (21%) as compared to those treated with EFV plus either two or three NRTIs (11%; *p* < 0.001) [21]. The randomized, double-blind MERIT study compared the efficacy and tolerability of maraviroc with EFV in treatment-naive patients infected with R5 HIV-1, with both treatment groups also receiving zidovudine and lamivudine [22]. At 48 weeks, maraviroc did not show non-inferiority compared with EFV for the primary endpoint of a HIV viral load <50 copies/mL (65.3% *vs.* 69.3%; lower limit of one-sided 97.5% CI–10.9%). In addition, more patients discontinued in the maraviroc compared with the EFV arm due to lack of efficacy (11.9% *vs.* 4.2%). The 004 study compared the efficacy and tolerability of raltegravir *vs.* EFV, both combined with tenofovir and lamivudine [23]. At 96 weeks, the raltegravir and EFV groups exhibited similar rates of viral suppression, with 83% and 84% of patients, respectively, achieving HIV viral load <50 copies/mL by intent-to-treat analysis. In the STARTMRK study, raltegravir-based combination therapy was found to be non-inferior to that of EFV through 156 weeks [24,25,26], but after 240 weeks raltegravir/tenofovir/emtricitabine seemed to have superior efficacy compared with EFV/tenofovir/emtricitabine [27]. Of note, in the SINGLE study which evaluated the integrase inhibitor dolutegravir, the proportion of patients having HIV-1 RNA <50 copies/mL was significantly higher in the abacavir/lamivudine/dolutegravir group than in the tenofovir/emtricitabine/EFV group (88% *vs.* 81%; *p* = 0.003) [28]. However, despite high levels of treatment success, resistance to EFV develops readily: resistance mutations arise in 6%–8% of patients treated with EFV plus two NRTIs for 2–3 years, with K103N being by far the most common single mutation. Other substitutions observed in Phase II trials include V108I, P225H or L100I, K101E, K101Q, Y188H, Y188L, G190S, G190A, and G190E.

## 7. Etravirine

ETR is a potent diarylpyrimidine (DAPY) analog which shows *in vitro* activity against a broad range of HIV-1 groups and subtypes, including strains that exhibit resistance to EFV and NVP [29]. Compared to EFV and NVP, ETR may also have a higher genetic barrier to resistance because at least two mutations are required to confer resistance. Mutations associated with ETR resistance selection *in vitro* include E138K, Y181C/I, V179F, G190E and M230L [29]. ETR efficacy was evaluated in adult treatment-experienced HIV-1 infected patients in two large multinational Phase III, 96 week, randomized, double-blind, placebo-controlled, clinical trials, DUET-1 and DUET-2 [30,31]. At entry, eligible participants must have had prior ART exposure for at least eight weeks with evidence of viral replication (plasma viral load of >5000 copies/mL). Furthermore, all participants were infected with HIV-1 strains that contained three or more primary protease inhibitor resistance mutations and at least one NNRTI resistance mutation. The primary endpoint of the study was the proportion of participants reaching an undetectable viral load (<50 copies/mL) at 24 weeks. A significantly larger portion of patients receiving optimized background therapy (OBT) plus ETR compared with OBT plus placebo reached an undetectable viral load at 24 weeks (56% *vs.* 39% in DUET-1, *p* < 0.01 and 62% *vs.* 44% in DUET-2, *p* < 0.001, respectively). A comprehensive analysis of study-entry resistance data from the DUET studies identified 17 ETR resistance-associated mutations, namely: V90I, A98G, L100I, K101E/H/P, V106I, E138A, V179D/F/T, Y181C/I/V, G190A/S, and M230L [32]. Importantly, the superior and durable virologic response for the OBT/ETR arm of the DUET trials was also observed at weeks 48 and 96, respectively. At week 96, virologic failure occurred in 93 (15.5%) of ETR-treated participants (compared with 170 (28.1%) placebo-treated participants) [33]. In the 93 failures, the most commonly emerging RT mutations were: V179F (16.1%), V179I (14.0%), Y181C (10.8%), V108I (8.6%) and K103N (7.5%). L74V, L100I, K101E, and M184V (6.5%), and E138G, E138Q, Y181I, V189I, E297K, and N348I (5.4%) also emerged. Of the emerging mutations, only five (L100I, K101E, V179F, Y181C, and Y181I) were previously identified as ETR resistance associated mutations. Based on 48-week data from the DUET-1 and DUET-2 trails, ETR was approved by the FDA for use in treatment-experienced adults who are experiencing virologic failure with HIV-1 strains resistant to an NNRTI and other antiretroviral agents. Of note, Ruxrungtham *et al.* reported that in a protease inhibitor-naïve population, with baseline NRTI and NNRTI resistance and NRTI recycling, TMC125 was not as effective as first use of a protease inhibitor due to baseline NRTI and NNRTI resistance [34].

## 8. Rilpivirine

Like ETR, RPV is a DAPY analog that shows potent activity against wild-type HIV-1 group M isolates (0.07 to 1.01 nM) and group O isolates (EC_50_ values range from 0.07 to 8.45 nM) [35]. In *in vitro* experiments, RPV shows a resistance profile and a genetic barrier to the development of resistance comparable to those of ETR [35]. NNRTI resistance associated mutations that emerged in HIV-1 under selective pressure from RPV included combinations of V90I, L100I, K101E, V106A/I, V108I, E138G/K/Q/R, V179F/I, Y181C/I, V189I, G190E, H221Y, F227C, and M230I/L. In humans, RPV efficacy was established in the THRIVE (TMC278 against HIV, in a once daily RegImen *vs* Efavirenz) and ECHO (Early Capture HIV Cohort Study) studies [36,37]. These two Phase III, multinational, double-blinded, randomized, placebo-controlled, non-inferiority studies compared the efficacy of RPV *vs.* EFV in combination with two NRTIs. In both studies, RPV was found to be non-inferior to EFV in treatment-naïve participants. However in both trails, RPV-treated participants had higher rates of virologic failure than EFV-treated participants (10% *vs.* 6%), particularly in individuals with pre-treatment HIV-1 viral loads >100,000 copies/mL (17% virologic failure in the RPV arm *vs.* 7% in the EFV arm). Resistance analyses demonstrated that the most common NNRTI-resistant mutation that emerged when participants failed RPV was E138K, typically in combination with M184I or M184V/I mixtures. Viral fitness studies have suggested that E138K may compensate for the fitness deficits of both M184I and M184V and restore the replicative capacity of viruses containing M184I/V [38,39]. However, other work suggests that viruses containing both the E138K and M184I mutations do not have high replicative fitness [40]. Other mutations detected in patients with demonstrable resistance in the pooled ECHO and THRIVE clinical trials included K101E, H221Y, V90I, Y181C, V189I, L100I, V179I, E138Q and F227C. Collectively, at least 17 single substitutions in HIV-1 RT (L100I, K101E/P, E138A/G/K/Q/R, V179L, Y181C/I/V, Y188L, H221Y, F227C, and M230I/L) have been associated with a decreased virologic response to RPV. Interestingly, we recently found that the E138A substitution occurs more frequently in subtype C (range: 5.9%–7.5%) than B (range: 0%–2.3%) sequences from both treatment-naïve and -experienced individuals (*p* < 0.01) in four independent genotype databases [41]. Importantly, there is a documented case report of an HIV-1-infected individual who did not respond to RPV/tenofovir/emtricitabine, apparently due to the presence of a baseline E138A mutation [42]. As such, E138A could impact treatment strategies that include RPV in geographic areas where subtype C infection is prevalent.

## 9. Long Acting Rilpivirine

A parentral, long-acting form of RPV (RPV-LA) has been developed with the goal of improving treatment adherence and for potential use as a PrEP agent [43]. A RPV-LA pre-clinical proof-of-concept study was conducted in mice and dogs, which showed sustained concentrations of the drug for over three weeks and three months, respectively [44]. These encouraging results led to at least two phase I clinical pharmacokinetic studies in which RPV-LA was generally well tolerated, and was shown to achieve sustained concentrations of the drug in the plasma and genital tract tissues [45,46]. These finding support its potential efficacy for use in PrEP. Additionally, RPV-LA in combination with the integrase inhibitor GSK1265744 is being assessed in an ongoing phase IIb study to determine whether they can maintain virologic suppression in HIV-infected individuals (ClinicalTrials.gov Identifier: NCT01641809).

## 10. Dapivirine

DAP is also a DAPY analog that demonstrates potent, dose-dependent inhibitory effects against a broad panel of HIV-1 isolates from different clades [47]. DAP was licensed to the International Partnership for Microbicides (IPM) for development as a topical microbicide. While DPV has been formulated as a gel and an intra-vaginal ring (IVR), the DAP IVR has moved ahead into late stage clinical evaluation. The IVR releases DAP over a 28 days period. After this time, the woman removes the ring and inserts a new one. The DAP IVR is currently in Phase III clinical testing in two separate trials. The first trial, called ASPIRE, is being conducted in several sub-Saharan countries, with completion expected in early 2015. The second trial, called the Ring Study (IPM-027) is a similar study is also expected to end in early 2015. DAP has also been co-formulated with the CCR5-blocker maraviroc in an IVR. This product is being evaluated in a Phase I safety, PK study (MTN-013). *In vitro*, DAP resistance correlates with the following mutations: V90I, L100I, K101E, V106I, V108I, E138K, E138G, Y181C, and Y188L [48].

## 11. MIV-150

MIV-150 is a PETT-urea analog with potent antiviral activity (EC_50_ of <1 nM). MIV-150 has been extensively evaluated in challenge models of HIV and the herpes simplex virus (HSV) when combined with carrageenan (the Carraguard product was found to be ineffective as a microbicide when administered alone), and zinc acetate [49,50]. Despite a lack in efficacy in preventing HIV-1 transmission, Carraguard was found to possibly prevent transmission of HPV Zinc salts have been found to possess activity against HSV-2 and HIV-1. The Population Council is developing several potential products combining MIV-150, carrageenan, and zinc acetate including a gel and IVR. A phase 1, double-blind, parallel, placebo-controlled, randomized study to evaluate the safety, pharmacokinetics, pharmacodynamics, and acceptability of MIV-150 containing microbicide gel formulation in HIV-seronegative women is currently ongoing (NCT02033109). In SHIV-RT infected rhesus macaques, MIV-150 resistance was associated with the K101E, V108I, E138A/K, K103N, V179A/I/L, Y181C/I and Y188H mutations [51].

## 12. Potential for Cross-Resistance between NNRTIs Used for Prevention and Treatment of HIV-1 Infection

In general, there is a high level of cross-resistance within the NNRTI class as a result of two mechanisms:

(i) Nearly all of the NNRTI resistance mutations are within or adjacent to the NNRTI-binding pocket. Indeed there is no evidence that any one mutation only confers resistance to a single agent: most NNRTI-resistance mutations reduce susceptibility to two or more NNRTIs (Table 1).
(ii) The genetic barrier to NNRTI resistance is low. Typically, EFV, NVP and RPV require only a single mutation to reduce clinical efficacy. ETR requires two mutations, but in certain circumstances (*i.e.*, Y181I/V) a single mutation may be sufficient.

In light of the high cross-resistance among the NNRTI class, there is a high likelihood that if an NNRTI-resistant variant is selected in an individual who became infected while using an NNRTI-containing PrEP or microbicide formulation, the efficacy of future 1st-line and salvage antiretroviral therapies may be highly compromised. Most concerning is the use of the structurally related DAPY analogs (DAP, RPV and ETR) which share over-lapping resistance pathways. Consequently, a better understanding of the resistance and cross-resistance profiles among the NNRTI class is important for predicting response to treatment, and surveillance of transmitted drug-resistance.

**Table 1:** Mutations associated with the NNRTIs discussed in this review.

NNRTI	V 90	L 100	K 101	K 103	V 106	V 108	E 138	V 179	Y 181	Y 188	G 190	H 221	P 225	F 227	M 230
NVP	I	I	EP	N	A/I/M	I		D/E/L	C/I/V	L/C/H	A/S/E		H	L/C	L
EFV	I	I	EP	N	A/I/M	I		D/E/L	C/I/V	L/C/H	A/S/E		H	L/C	L
ETR	I	I	EP		I		A/G/K/Q	D/E/F/I/T/L	C/I/V	L	A/S/E			C	L
RPV	I	I	EP		I		A/G/K/Q	D/E/F/I/T/L	C/I/V	L	A/S/E	Y		C	L
DAP	I	I	E			I	K		C	L					
MIV-150			E	N	I	I	A/K	A/I/L	C/I	H					
